# The Wnt/β-catenin signalling pathway in Haematological Neoplasms

**DOI:** 10.1186/s40364-022-00418-9

**Published:** 2022-10-13

**Authors:** Siwei Yu, Ruyue Han, Runliang Gan

**Affiliations:** Cancer Research Institute, Key Laboratory of Cancer Cellular and Molecular Pathology in Hunan Province, Hengyang Medical School, University of South China, 421001 Hengyang, Hunan P. R. China

**Keywords:** Wnt/β-catenin signalling pathway, Haematologic neoplasms, Leukaemia, Lymphoma, Multiple myeloma

## Abstract

Leukaemia and lymphoma are common malignancies. The Wnt pathway is a complex network of proteins regulating cell proliferation and differentiation, as well as cancer development, and is divided into the Wnt/β-catenin signalling pathway (the canonical Wnt signalling pathway) and the noncanonical Wnt signalling pathway. The Wnt/β-catenin signalling pathway is highly conserved evolutionarily, and activation or inhibition of either of the pathways may lead to cancer development and progression. The aim of this review is to analyse the mechanisms of action of related molecules in the Wnt/β-catenin pathway in haematologic malignancies and their feasibility as therapeutic targets.

## Introduction

Common haematologic malignancies include various types of leukaemia, malignant lymphoma, and multiple myeloma. Physico-chemical, biological and genetic factors play an important role in the development of malignant diseases of the haematological system. For example, people who are exposed to formaldehyde and radiation are 20 to 30 times more likely to develop leukaemia than the general population. Human T-lymphotropic virus type I causes T-cell leukaemia/lymphoma in adults, and more than 80% of patients with Burkitt’s lymphoma have significantly higher titres of EBV antibodies in their sera [1, 2].

With the continuous development of society, the occurrence of malignant neoplasms in the haematological system has also increased each year. In addition to genetic factors [3], the Wnt/β-catenin pathway serves as an important factor closely related to the development of malignant diseases, and its role in haematologic malignancies has received extensive attention and research by scientists and has been confirmed in various experiments.

In 1982, Nusse and Varmus isolated the first Wnt gene in the mouse genome, and subsequent studies found that ectopic expression of Wnt induced mammary carcinogenesis in mice and defects in insect embryonic development [4]. In the 40 years since, the role of the Wnt signalling pathway in embryonic development and cancer development and progression has been gradually revealed. In particular, the canonical Wnt pathway, also known as the Wnt/β-catenin signalling pathway, is involved in the development and progression of a range of cancers. The role of the Wnt signalling pathway was first demonstrated in colorectal cancer. In 1997, Kris Vleminckx et al. proposed that the tumour suppressor gene APC, which is associated with β-catenin as part of the classical Wnt signalling pathway, plays an important role in carcinogenesis in familial adenomatous polyposis [5]. In addition, new studies have found that MASTL induces colon cancer progression and chemoresistance by promoting Wnt/β-catenin signalling [6]. In a recent liver cancer-related study, Fu et al. proposed that Linc00210 drives Wnt/β-catenin signalling activation and liver tumour progression in a CTNNBIP1-dependent manner [7]. In another study, Wantae Kim et al. found that Wnt/β-catenin signalling activation inhibited HCC formation by suppressing the positive feedback loop between YAP/TAZ and Notch signalling [8]. In breast cancer, cell surface GRP78 and dermcidin cooperate to regulate breast cancer cell migration via Wnt signalling [9]. LncRNA PKMYT1AR promotes cancer stem cell maintenance in non-small-cell lung cancer by activating the Wnt signalling pathway [10]. AXIN1-295aa, a newly identified protein encoded by circAXIN1, promotes gastric cancer progression by activating the Wnt/β-catenin signalling pathway [11]. In melanoma, downregulation of RNF128 activates Wnt/β-catenin signalling to induce cellular EMT and increase stemness via CD44 and Cortactin ubiquitination [12].

Although exciting results about the Wnt classical signalling pathway have been obtained from studies of the aforementioned solid cancers, its roles are poorly understood in haematologic malignancies, likely because most haematologic neoplasms are not solid cancers, which makes research difficult. This review summarizes and outlines recent advances in the understanding of the Wnt/β-catenin signalling pathway in haematologic malignancies and explores potential therapeutic targets identified in recent years.

## The wnt signalling pathway

The Wnt signalling pathway is critical for human development. The Wnt family consists of at least 19 secreted glycoproteins with 22–24 conserved cysteine residues, and these proteins are associated with human development and disease occurrence [13]. The Wnt signalling pathway is divided into the canonical Wnt signalling pathway, which is β-catenin dependent, and the noncanonical Wnt signalling pathway, which is not dependent on β-catenin.

### The noncanonical wnt signalling pathway

The noncanonical Wnt signalling pathway includes (1) the planar cell polarity pathway, which is involved in the activation of JNK and cytoskeletal rearrangement; (2) the Wnt/ Ca^+^ pathway, which activates PLC and PKC [14]; and (3) intracellular pathways that regulate spindle orientation and asymmetric cell division.

### The canonical wnt signalling pathway

**Fig. 1:**
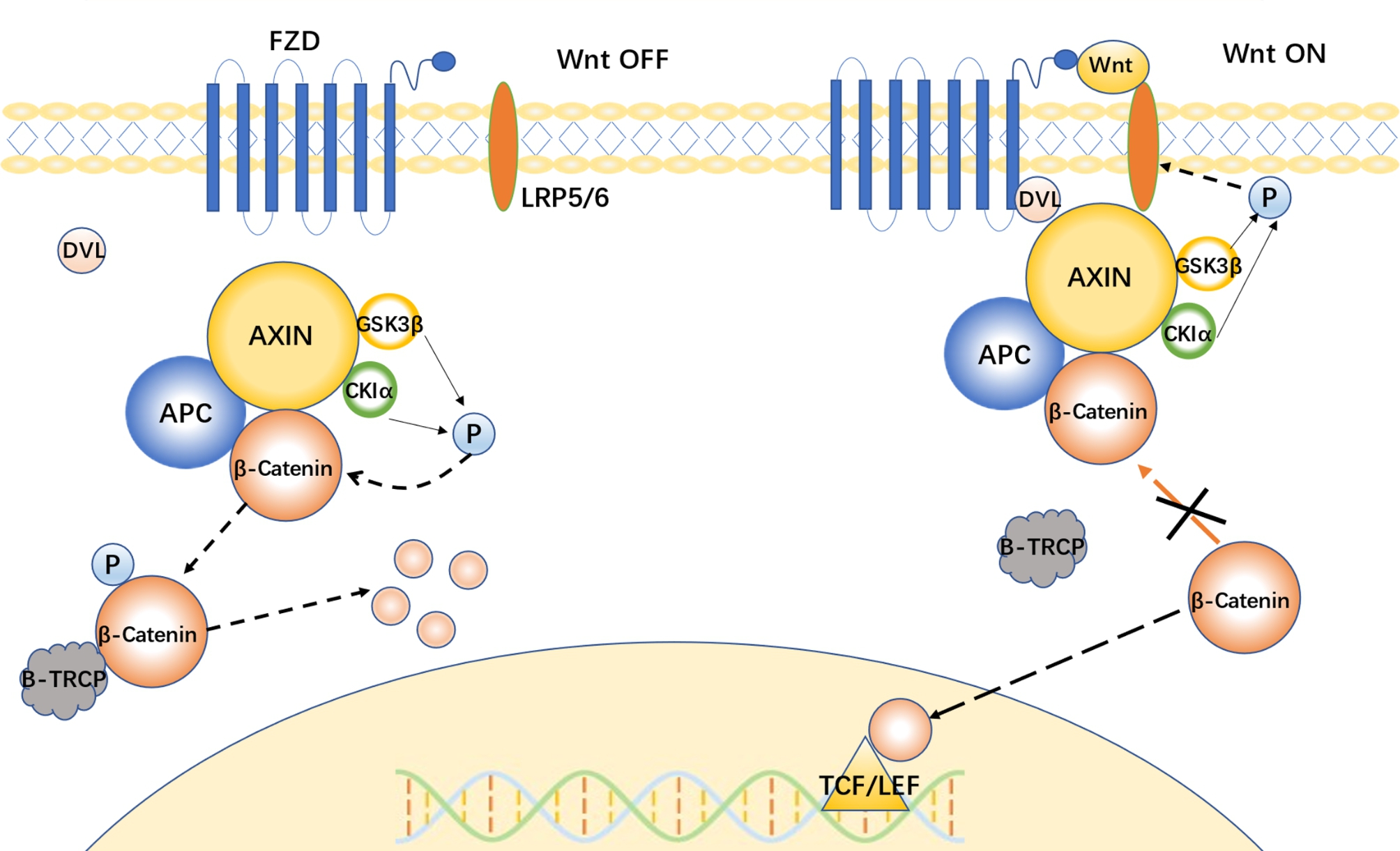
Regulation of the Wnt/β-catenin signalling pathway. (* As shown in the picture, Wnt signalling is divided into two states: on and off. In the off state, β-catenin in the cytoplasm is phosphorylated by the disruption complex with AXIN as the main scaffold and subsequently presented to the E3 ubiquitin ligase β-TrCP for ubiquitination and degradation. When Wnt ligands bind to FZD-LRP5/6 on the cell membrane, the disruption complex is recruited to the cell membrane. At this point, β-catenin cannot be phosphorylated and occupies the disruption complex, resulting in the accumulation of newly generated β-catenin in the cytoplasm, which then enters the nucleus to regulate TCF/LEF transcription by the action of related molecules)

Here, we focus on the canonical Wnt signalling pathway [15]. β-Catenin, a component of calmodulin-based adhesion junctions [16], is an extremely important effector in the canonical pathway. A destruction complex exists in the cytoplasm, consisting mainly of AXIN, the tumour suppressor gene APC, GSK3β and CK1α [17]. In the absence of Wnt proteins, the so-called Wnt-off state, AXIN acts as a scaffolding protein that binds β-catenin, and AXIN also binds GSK3β, CK1α, and APC. CK1α and GSK3β can sequentially phosphorylate β-catenin [18], and APC then ensures that phosphorylated β-catenin is not dephosphorylated by PP2A and later phosphorylation of β-catenin [19]. Phosphorylation of β-catenin exposes a binding site for the E3 ubiquitin ligase β-TrCP, and β-catenin is thus ubiquitinated and degraded [20], as such, it is unable to enter the nucleus to initiate downstream gene transcription. When Wnt proteins are present, the so-called Wnt-on state, the Wnt ligands bind to FZD and LRP5/6 on the cell membrane [21, 22]. The binding of Wnt to FZD exposes an intracellular binding site for DVL in FZD. The DEP and PDZ regions on DVL can thus interact with this site [23]. Meanwhile, the DIX region of DVL can form a dimer with the DAX region of AXIN[24], so the destruction complex is recruited to the FZD-Wnt-LRP5/6 complex on the membrane. Subsequently, GSK3β and CK1α no longer phosphorylate β-catenin and instead phosphorylate the five phosphorylatable groups on LRP5/6 [25]. When phosphorylated, any of these groups can become a binding site for AXIN [26, 27]. Then, LRP5/6 can recruit the destruction complex and complete the phosphorylation of the other remaining groups [28], which initiates a positive feedback loop. All of these factors result in functional β-catenin accumulating in the cytoplasm and entering the nucleus with the assistance of relevant molecules. Thereafter, β-catenin regulates the transcription of transcriptional regulators such as TCF/LEF and genes that are ultimately targeted by Wnt [29] (Fig. 1). In addition, it has been shown that GSK3β and CK1α also phosphorylate AXIN and APC, leading to increased binding of AXIN and APC to β-catenin, thereby enhancing the phosphorylation of β-catenin [18, 30, 31].

## The role of the Wnt/β-catenin signalling pathway in haematopoietic stem cell development and cancer stem cell formation

Haematopoietic stem cells are the stem cells of the adult blood system and have the functions of self-renewal and differentiation into various mature blood cells. During their differentiation, adverse intrinsic or extrinsic stimuli may lead to the development of malignant diseases of the blood system [32], and the canonical Wnt/β-catenin signalling pathway plays an important role in regulating the process of cell proliferation and differentiation. The Wnt/β-catenin pathway promotes early engraftment of foetal haematopoietic stem/progenitor cells [33]. The expression of structurally active β-catenin in lymphoid and myeloid cells can contribute to the formation of immature cells with multiple differentiation potentials, which suggests a role for Wnt signalling in maintaining the undifferentiated state of haematopoietic stem cells [34]. Studies have shown that the self-renewal of haematopoietic stem cells is hindered when Wnt signalling is inhibited by DKK1, which is an inhibitor of Wnt signalling. DKK1 binds to the Wnt coreceptor LRP5/6 and desensitizes cells to canonical Wnt ligand signalling [35]. Wnt signalling is required for normal haematopoietic cell function and can contribute to the differentiation of stem cells into a variety of different phenotypes [36, 37]. Deficiency of Wnt3a restrains Wnt/β-catenin signalling, irreversibly impairing haematopoietic stem cell self-renewal and leading to defects in progenitor cell differentiation [38, 39]. The role of Wnt signalling in HSCs is related to its degree of activation. In a study by Luis et al., intermediate and higher levels of activation of the canonical pathway impaired HSC proliferation capacity. Only mildly increased Wnt signalling levels enhanced HSC repopulation capacity [40]. Wang’s study also showed that levels of Wnt signalling activity affect the sensitivity of HSCs to DNA damage-induced depletion in a mouse model [41].

In addition, CBP and P300 are essential for the self-renewal and normal differentiation of haematopoietic stem cells, and mutations in P300 and CBP are found in many malignancies. In acute myeloid leukaemia, chromosomal translocations affecting CBP and, less commonly, P300 are found. Mouse models have also confirmed that P300 and CBP are inhibitors of haematologic neoplasm formation[42–44]. The apoptosis suppressor gene survivin is tumour-specific, and transcription of the survivin gene is regulated by TCF, a downstream molecule of Wnt/β-catenin, in a CBP-dependent manner. When CBP is inhibited, the β-catenin/CBP interaction is disrupted. The expression of c-myc, another well-known TCF/β-catenin target, increases with the recruitment of P300 to the c-Myc promoter[42, 45]. Furthermore, oxidative stress in cells can activate the Wnt/β-catenin/MYC/Sox2 axis to enhance the oncogenicity and stemness of ALK-positive mesenchymal large-cellymphoma [46].

Cancer stem cells, by functional definition, are a small fraction of cancer cells with stem cell-like functions in neoplastic tissue that maintain neoplastic cell proliferation, invasion, and metastasis. Cancer stem cells make up a very small percentage of neoplastic cells and have their own unique and specific molecular markers. Compared with neoplastic cells, which are negative for specific molecular markers, cancer stem cells have a significantly enhanced ability to invade and metastasize. For example, CD133 + hepatocellular carcinoma cells have higher clonogenicity in vivo and tumorigenicity in vitro than their CD133- counterparts [47]. ALDH is widely used as a CSC marker in many types of cancer, including colon [48], breast [49], ovarian [50], bladder [51], and prostate cancer [52]. The initial discovery of cancer stem cells was in leukaemia, and the cells were first detected by John Dick et al. [53]. The conversion of normal haematopoietic stem cells into cancer stem cells is caused by various factors and is the main reason for the occurrence of malignant haematological disease as well as recurrence. For example, the mixed-lineage leukaemia gene can be altered form fusion genes. The fusion genes *Mll-af9*, *Hoxa9* and *meisla* can induce the transformation of haematopoietic stem cells into leukaemic stem cells. In-depth studies have revealed that fusion genes can induce differentiated progenitor cells to become LSCs by activating self-renewal signalling pathways, such as the β-catenin signalling pathway [54, 55]. The Wnt/β-catenin pathway plays an important role in the maintenance, proliferation, and differentiation of haematopoietic stem cells. It has been shown that Wnt/β-catenin signalling can be selectively regulated by the RUNX1 promoter in leukaemic cells and human haematopoietic progenitor cells. The P1-RUNX1 distal promoter is a direct transcriptional target of Wnt/β-catenin signalling and may play an important role in the transformation of normal haematopoietic cells or stem cells into malignant stem cells [56]. Bmi1, as a negative regulator of the canonical Wnt signalling pathway in haematopoietic stem and progenitor cells, can enhance HSC self-renewal, thereby improving the outcomes of HSC transplantation [57]. There is limited information on the role of these factors in the transformation of haematopoietic stem cells into cancer stem cells, but a study showed that the Wnt/β-catenin signalling pathway is required for the self-renewal of LSCs derived from either haematopoietic stem cells or more differentiated granulocyte-macrophage progenitors [55]. Research aimed at understanding the transformation of haematopoietic stem cells into malignant stem cells by activation of the Wnt canonical pathway is promising. Targeting Wnt/β-catenin to successfully inhibit the transformation of haematopoietic stem cells would be applicable across disciplines.

## The role of the Wnt/β-catenin signalling pathway in leukaemia

Leukaemias are a group of malignant clonal diseases of haematopoietic stem and progenitor cells. Leukaemic cells have strong proliferative capacity that can inhibit normal haematopoietic function, but they stop developing at different stages because their proliferation is uncontrolled, they have impaired differentiation and impaired apoptosis, and they lack normal functions. Leukaemias are divided into two categories, acute and chronic, according to the degree of differentiation and maturation of the cells. Acute leukaemia is divided into acute lymphoblastic leukaemia and acute myeloid leukaemia. Chronic leukaemia is divided into chronic myeloid leukaemia, chronic lymphocytic leukaemia, and there are other rare types of leukaemia. Several mechanisms can lead to overactivation of the Wnt/β-catenin signalling pathway that causes leukaemia, such as abnormal expression of Wnt protein and dysfunction of the destruction complex (Table 1).

**Table 1:** Wnt/β-catenin-related molecular mechanisms in leukaemia

Factor	Target (location)	Disease	Effect
P13K/AKT	GSK3β	ALL	decrease drug resistance of LSCs [58]
LY294002/P13K/AKT	GSK3β	T-ALL	decrease the effects of P13K/AKT signalling [59]
MYCN/DKK3	β-catenin	B-ALL	promote the proliferation of cancer cells [68]
FLT3	β-catenin	AML	induce the expression of c-Myc [70]
FLYWCH1	β-catenin (nucleus)	AML	inhibit cell cycle progression at G0 [71]
DIXDC1	β-catenin (nucleus)	AML	promote the proliferation of cancer cells [72]
CEBPA/PLIN2	β-catenin	CML	promote the development of CML [74]
RNA LOC101928834/FRAT2	GSK3β	AML/MM	promote the proliferation of cancer cells [83]

The Wnt/β-catenin pathway, like the PI3K-Akt pathway, is very commonly mutated in malignant diseases. LSCs are the main component responsible for resistance to leukaemia treatment. In ALL, Akt can activate β-catenin by inhibiting GSK3β. Reducing the immune resistance of LSCs by inhibiting Akt-activated β-catenin is a good strategy for cancer treatment and preventing immune evasion [58]. LY294002, a P13K inhibitor, reversed the action of the PI3K-Akt pathway by inhibiting the formation of p-GSK3β [59].

### The role of the Wnt/β-catenin signalling pathway in T-cell acute lymphoblastic leukaemia

In T-ALL, the PI3K/Akt/mTOR pathway increases HIF-1α activity in hypoxic cancer cells [60]. Moreover, β-catenin potentiates the activity of endogenous HIF-1α under hypoxic conditions [61]. However, HIF-1α can upregulate β-catenin activity and activate the PI3K/Akt/mTOR pathway in hypoxic T-ALL cells [62, 63], creating a vicious cycle. Combined targeting with the CBP/β-catenin transcriptional inhibitor ICG001 and the PI3K inhibitor ZSTK474 downregulated the proliferation, survival and clonogenic activity of T-ALL cells and induced apoptosis. This apoptosis induction was associated with downregulation of the Wnt/β-catenin and PI3K/Akt/mTOR pathways [64]. The combination of these inhibitors was more effective than either single agent. This result suggests that targeting both the Wnt/β-catenin signalling pathway and the P13K/AKT pathway is significant for the treatment of leukaemia.

### The role of the Wnt/β-catenin signalling pathway in B-cell acute lymphoblastic leukemia

MYCN belongs to a small gene family that also includes the c-myc and l-myc genes [65]. MYCN was found to negatively regulate DKK3 at the transcriptional level. DKK3 is an endogenous inhibitor of the canonical Wnt signalling pathway [66]. Downregulation of DKK3 has been correlated with β-catenin accumulation. However, the underlying molecular mechanisms remain to be fully understood [67]. After silencing MYCN expression, the level of DKK3 was restored, which inhibited Wnt/β-catenin expression and thus reduced cell proliferation and increased apoptosis and G1 phase arrest [68].

### The role of the Wnt/β-catenin signalling pathway in acute myeloid leukaemia

In AML, FLT3 mutations are often found. FLT3 is a type 3 receptor tyrosine kinase. It is the most commonly mutated gene in acute myeloid leukaemia [69]. Aberrant FLT3 signalling increases the nuclear localization and transcriptional activity of β-catenin, which induces the expression of the downstream gene c-myc and leads to the development of AML. The combination of chemotherapy with a tyrosinase inhibitor and the β-catenin inhibitor C-82 significantly inhibited the expression of c-myc, which has positive implications for treatment [70]. In a study of AML by Amany Almars et al., a newly discovered protein, FLYWCH1, was identified as a negative regulator of the Wnt/β-catenin pathway; FLYWCH1 was found to directly bind to nuclear β-catenin, and when it was highly expressed, it suppressed the expression of c-myc, CyclinD1 and c-Jun, downstream target genes of Wnt/β-catenin signalling, while blocking cells in G0 phase [71]. Xin et al. investigated the relationship between the DIXDC1 gene and the Wnt/β-catenin pathway in AML. DIXDC1 is a protein containing both DIX and MTH structural domains. It is a positive regulator of the Wnt pathway. Dysregulation of DIXDC1 is closely associated with the development and progression of a variety of cancers and can activate the PI3K/AKT signalling pathway as well as the Wnt signalling pathway, which regulates β-catenin entry into the nucleus [72]. In AML, DIXDC1 overexpression promotes the proliferation of AML cells, accelerates cell cycle progression and reduces apoptosis. DIXDC1 knockdown reduces the expression of Wnt/β-catenin target genes, including CyclinD1 and c-myc, while overexpression of DIXDC1 has the opposite effect. Knockdown of the β-catenin gene reverses the oncogenic effect of DIXDC1 to some extent, suggesting that DIXDC1 may promote the growth of acute myeloid leukaemia cells through upregulation of the Wnt/β-catenin signalling pathway. DIXDC1 may be a therapeutic target for AML [73].

### The role of the Wnt/β-catenin signalling pathway in chronic myelogenous leukaemia

CEBPA is a key regulator of myeloid differentiation and regulates many protein-coding genes. CEBPA upregulates the expression of the lncRNA PLIN2, and PLIN2 promotes the occurrence and development of CML by upregulating the expression of β-catenin, providing a theoretical basis for targeting the CEBPA/PLIN2 axis for the treatment of CML [74].

Experiments have confirmed that transferrin conjugated to doxorubicin has significantly increases cytotoxicity compared with the free drug. Transferrin has successfully been used as a carrier molecule to deliver doxorubicin to resistant leukaemia cells [75]. Sevoflurane, a volatile anaesthetic, negatively regulates CD34 + CML stem/progenitor cell biological properties. Sevoflurane effectively augments dasatinib efficacy in CML cell lines and stem/progenitor cells. These findings reveal that β-catenin is the target of sevoflurane and that sevoflurane decreases β-catenin activity [76]. Anisomycin can inhibit CML cells of all developmental stages and is more effective in combination with conventional chemotherapeutic agents, but as a monotherapy, it is less effective against the blast phase of CML [77]. It may have potential for CML treatment. In other studies, β-catenin overexpression affected highly proliferating CD4 + CD8 + double-positive thymocytes and caused aberrant activation of c-myc, leading to the development of Notch-independent leukaemia [78].

### The role of noncoding RNAs associated with Wnt/β-catenin signalling in leukaemia

Noncoding RNAs, including miRNAs and lncRNAs, have been popular research topics for many years and have been found to play an important role in leukaemia in recent studies. For example, the miR-29b/Sp1/FUT4 regulatory axis may promote the progression of acute myeloid leukaemia through fucosylation and CD44-mediated Wnt/β-catenin signalling. Glycosylation is a very common form of posttranslational protein modification, and fucosylation is one of the most important types of glycosylation in cancer. Aberrant fucosylation of proteins is a well-known hallmark of cancer [79, 80]. In AML, hsa_circ_0121582, as a sponge of miR-224, can reduce the inhibition of GSK3β induced by miR-224. Thus, it can decrease the accumulation of β-catenin in blasts and inhibit the proliferation of leukaemia cells [81].

The long noncoding RNA LOC101928834 is linked to the Wnt/β-catenin signalling pathway via FRAT2. FRAT2, a weak Wnt signalling activator, binds to GSK3β and promotes the activation of Wnt signalling, leading to the expression of a series of downstream genes [82]. FRAT2 promotes cell proliferation and cell cycle progression in vitro, leading to the development of myelodysplastic syndromes and AML [83]. The lncRNA HOTAIR was found to inhibit the immune rejection of mouse leukaemia L1210 cells by activating the Wnt/β-catenin signalling pathway in a mouse model of leukaemia [84]. The exact molecular mechanism remains unclear. However, it has been shown that HOTAIR inhibits the expression of WIF-1, an inhibitor of the Wnt/β-catenin signalling pathway, and activates the Wnt signalling pathway [85].

In conclusion, by targeting various factors related to the Wnt/β-catenin pathway, these newly discovered molecules affect the proliferation and/or apoptosis of leukaemic cells in different ways. Strategies targeting these molecules to treat leukaemia are worthy of further study, and some of them have shown promising therapeutic effects in clinical trials.

## The role of the Wnt/β-catenin signalling pathway in lymphoma

Lymphomas are malignant neoplasms of the blood system that originate in lymph nodes and extranodal lymphoid tissue. They are histopathologically classified into Hodgkin’s lymphoma and non-Hodgkin’s lymphoma. Lymphoma is a large group, and its pathogenesis varies. Physicochemical factors, immune factors, and genetic factors all play an important role in lymphoma morbidity. Despite the wide variety of lymphomas, studies of the Wnt/β-catenin signalling pathway in relation to lymphoma have focused on diffuse large B-cell lymphomas (Table 2).

**Table 2:** Wnt/β-catenin-related molecular mechanisms in lymphoma

Factor	Target	Disease	Effect
Smad5-AS1/miR-135B-5p	APC	DLBCL	restrain the proliferation of cancer cells [86, 87]
CircRNA-APC/miR-888	APC	DLBCL	restrain the proliferation of cancer cells [88]
MYC/FIRRE	nuclear translocation of β-catenin	DLBCL	promote the generation of cancer cells [91]
FOXM1/lncRNAOR3A4	nuclear translocation of β-catenin	DLBCL	promote the proliferation and generation of cancer cells [92]
TIMD4	nuclear translocation of β-catenin	DLBCL	promote proliferation and restrain apoptosis [93]
Foxp1/CBP	β-catenin	DLBCL	promote transcription of β-catenin [94]

Research has found that the long noncoding RNA Smad5-AS1 can act as a competitive RNA for miR-135B-5p to upregulate APC expression and inhibit the proliferation of diffuse large B cell lymphoma cells [86, 87]. CircRNA-APC also targets APC. CircRNA-APC is a circular RNA that originates from the reverse splicing of APC exon 7 to exon 14. CircRNA-APC inhibits Wnt/β-catenin signalling by interacting with TET-1 and miR-888, thereby suppressing the proliferation of diffuse large B-cell lymphoma cells. CircRNA-APC expression is downregulated in DLBCL cells. In a gain-of-function study, high expression of CircRNA-APC inhibited DLBCL cell proliferation in vitro and neoplasm growth in vivo. Cytoplasmic circRNA-APC acts as a sponge for miR-888, attenuating the repressive effect of miR-888 on APC and upregulating APC after transcription; APC thus can form the destruction complex to phosphorylate and degrade β-catenin in the cytoplasm [88]. Epstein‒Barr virus is a common herpesvirus. Most people worldwide have been infected with it, and infections are usually asymptomatic. However, an increasing number of studies have shown that Epstein‒Barr virus infection is closely related to the occurrence of lymphoma [89]. Mechanistic analysis showed that circEAF2 specifically targets EBV-encoded miR-BART19-3p, upregulates APC, suppresses downstream β-catenin expression, and counteracts EBV + DLBCL progression [90].

By regulating the nuclear translocation of β-catenin, long noncoding RNAs play a substantial role in the development of lymphoma. MYC expression is upregulated in DLBCL cells and positively regulates the lncRNA functional intergenic repeat RNA elements (FIRRE). When FIRRE is activated, it promotes diffuse large B-cell lymphoma by inducing nuclear translocation of β-catenin. When the FIRRE gene is knocked down, cell proliferation is reduced, and apoptosis is increased [91]. FOXM1, a transcription factor, has been reported to promote the transcription of a variety of lncRNAs. In one study, FOXM1 was found to induce upregulation of lncRNAOR3A4. OR3A4 can lead to the development and progression of diffuse large B-cell lymphoma by promoting nuclear translocation of β-catenin [92].

Other molecular pathways also regulate nuclear translocation. T-cell immunoglobulin and mucin-containing structural domain 4 is an important factor in various malignant diseases. TIMD4 exerts its function by promoting the nuclear translocation of β-catenin [93]. The results of experiments in DLBCL showed that TIMD4 was overexpressed in DLBCL tissues, and increased TIMD4 expression was significantly associated with poor prognosis of DLBCL patients. Knockdown of TIMD4 blocked cell growth and accelerated apoptosis, while upregulation of TIMD4 promoted cell proliferation and inhibited apoptosis.

In DLBCL, the transcription factor Foxp1 promotes acetylation of β-catenin by CBP, which increases the transcription of β-catenin/TCF7L2 target genes. Thus, Foxp1 is an active enhancer of the Wnt/β-catenin signalling pathway, but the exact mechanism by which Foxp1 recruits CBP is unclear [94]. By differentially expressed gene screening and bioinformatics analysis, a pair of highly related HUB genes, FBN1 and TIMP1, were identified in DLBCL. The FBN1/TIMP1 interaction promotes DLBCL cell migration and regulates the Wnt signalling pathway [95].

Moreover, some new discoveries have built on previous findings. GPNMB is a transmembrane glycoprotein that is highly expressed in a melanoma cell line and has low metastatic properties [96]. It has been confirmed to be related to the pathogenesis of tumours. In DLBCL, GPNMB activates the Wnt/β-catenin signalling pathway by targeting YAP1. This finding emphasizes the key role of GPNMB in the tumorigenesis of DLBCL, which may provide a new strategy for DLBCL therapy [97]. In another study, miR-361-3p was found to inhibit Wnt/β-catenin protein signalling by targeting Wnt-10A. Wnt-10A is an important factor in the suppression of lymphoma development. The miR-361-3p/Wnt10A axis may be a target for lymphoma treatment [98].

## The role of the Wnt/β-catenin signalling pathway in multiple myeloma

Multiple myeloma is a malignant proliferative disease of plasma cells. Abnormal proliferation of clonal plasma cells in the bone marrow and secretion of monoclonal immunoglobulins or their fragment M proteins results in damage to organs or tissues. The natural course of MM is highly heterogeneous, with a median survival of approximately 3–4 years, and few patients survive for more than 10 years. Numerous factors affect the prognosis of MM patients, and the Wnt/β-catenin pathway plays a substantial role in the development of multiple myeloma. The utility of targeting this pathway to treat MM has been validated in various trials [3, 99] (Table 3).

**Table 3:** Wnt/β-catenin-related molecular mechanisms in multiple myeloma

Factor	Target	Disease	Effect
miR-744-5p/SOX12	β-catenin	MM	promote apoptosis and inhibit proliferation [102, 103]
miR-30-5P/BCL9	Wnt	MM	restrain the proliferation and migration of cancer cells [106]
miR-128-3p/PLAGL2	β-catenin	MM	restrain the proliferation and migration of cancer cells [108]
RRM2	GSK3β	MM	induce apoptosis [110]
PCDH10/AKT	GSK3β	MM	promote proliferation and restrain apoptosis [111]
Lycorine	β-catenin	MM	restrain MMSCs [112]
DHA	β-catenin	MM	induce autophagy [113]
resveratrol	nuclear translocation of β-catenin	MM	restrain the proliferation and migration of cancer cells [114]
CGK012	β-catenin	MM	restrain the proliferation of cancer cells [115]

SOX12 is closely associated with many types of human cancers, such as osteosarcoma [100] and gastric cancer [101]. In MM, SOX12 has been found to promote the growth of MM cells by increasing the expression of functional β-catenin in the cytoplasm and upregulating the Wnt/β-catenin signalling pathway [102]. miR-744-5p directly regulates SOX12 by binding to the 3’-UTR of SOX12. SOX12 gene silencing by miR-744-5p significantly decreased the expression of β-catenin, increased the apoptosis rate of MM cells and inhibited the proliferation of MM cells [103].

MiR-135b was found to play a facilitating role in MM. Overexpression of miR-135b increased the expression of β-catenin, Wnt-3a and cyclin D1, while the expression of GSK3β and CK1α was decreased. Versican protein, mainly found in the extracellular matrix, is a large chondroitin sulfate proteoglycan that plays an important role in cell adhesion, migration, proliferation and differentiation. Wnt/β-catenin signalling regulates versican expression by acting on the upstream promoter of the versican gene [104]. Versican was found to be upregulated in MM, and silencing Versican reversed the effects of activating the miR-135b-mediated Wnt/β-catenin signalling pathway on the proliferation, migration, invasion and apoptosis of MM cells [105]. In addition, miR-30-5P inhibits the proliferation and migration of MM cells by targeting the 3’UTR of BCL9, a key coactivator of the Wnt/β-catenin signalling pathway, and downregulating the transcriptional activity of BCL9 and Wnt [106]. Cyclic RNA protein tyrosine kinase 2 promotes the proliferation and migration and inhibits the apoptosis of multiple myeloma cells through activation of the microRNA-638-mediated MEK/ERK and Wnt/β-catenin signalling pathways [107]. The pleomorphic adenoma gene LIK2 enhances β-catenin expression and nuclear translocation by decreasing β-catenin phosphorylation and upregulates CyclinD-1 expression to promote MM [108]. In addition, miR-128-3p specifically silences PLAGL2 and inhibits neoplastic cell proliferation and migration. Interestingly, the long noncoding RNA HCP5 was found to be able to sponge miR-128-3p, leading to PLAGL2 overexpression that activated the classical Wnt pathway [109].

MM development can be disrupted by modulating GSK3β, an important phosphatase in the Wnt/β-catenin pathway, to promote or inhibit Wnt signalling. A study showed that RRM2 knockdown activated the phosphorylation of GSK-3β, decreased the expression of β-catenin, and significantly downregulated the expression levels of c-myc and cyclin D1, thereby inducing apoptosis [110]. PCDH10 also promotes GSK3β expression by inhibiting AKT, thus inhibiting β-catenin activation. PCDH10 is generally deficient in MM. Restoration of PCDH10 inhibited the nuclear localization of β-catenin, the activity of LEF/TCF, and the expression of BCL9 and AKT while upregulating the expression of GSK3β [111].

Several botanical preparations have shown promising effects in MM. The lycoris bulb extract lycorine inhibits the Wnt/β-catenin pathway by decreasing β-catenin protein levels, thereby reducing the number of bone marrow mesenchymal stem cells. In addition, lycorine reduces the increase in the proportion of ALDH1 + cells induced by bortezomib. BZM in combination with lycorine has a synergistic effect against myeloma cells. It was also found that lycorine has a similar inhibitory effect on the MMSC properties of BZM-resistant MM cells and primary CD138 + plasma cells [112]. Recent studies have shown that dihydroartemisinin can induce autophagy in MM cells by inhibiting β-catenin, which has guiding implications for the application of artemisinin in the treatment of MM [113]. In addition, the phytopharmaceutical agent resveratrol inhibits the proliferation, migration and invasion of MM cells by suppressing the Wnt/β-catenin signalling pathway and the unfolded protein response. Experimental application of resveratrol increased β-catenin expression in the cytoplasm and decreased it in the nucleus, suggesting that it may act in a way that regulates β-catenin nuclear translocation [114]. The pyranocoumarin compound CGK012 inhibits the activation of WNT3a-CM-mediated transcription of β-catenin. CGK012 induces β-catenin phosphorylation at Ser33/Ser37/Thr41, leading to proteasomal degradation and reducing intracellular β-catenin levels [115].

## Therapies targeting the Wnt/β-catenin signalling pathway in haematological malignancies

The main traditional methods for treating cancers are radiotherapy and chemotherapy; however, they are not very effective, and they have obvious side effects, such as bone marrow suppression and gastrointestinal reactions. Therefore, it is necessary to find new, more effective and safe methods for the treatment of malignant haematological diseases. A large number of experiments have proven that it is feasible to treat malignant haematopoietic diseases by targeting the Wnt/β-catenin pathway. Some experiments have reported the effects of relevant drugs in animal models and clinical trials (Table 4).

**Table 4:** Therapy targeting the Wnt/β-catenin signalling pathway in haematological malignancies

Factor	Target	Disease	Clinical Trials
CWP232291	β-catenin	AML	NCT01398462 [116]
PRI-724	CBP/β-catenin	AML/CML	NCT01606579 [117]
Lawsone	FZD	ALL	[118]
WNT974	β-catenin	lymphoma	[119, 120]
PRMT5	Wnt/β-catenin and AKT/GSK3β	lymphoma	[121]
AV-65	β-catenin	MM	[122]
DAC/BZM	β-catenin/GSK3β	MM	[123]
BC2059	β-catenin	MM	[124]
panobinostat/Tegavivint	β-catenin	MM	[125]

Several clinical studies of strategies targeting the Wnt/β-catenin pathway for leukaemia treatment are underway.

CWP232291 is a small-molecule inhibitor of Wnt signalling that causes degradation of β-catenin via apoptosis induction through endoplasmic reticulum stress activation. In AML, CWP232291 has no apparent organ toxicity, and its mechanism of action does not rely on nonspecific myelosuppression, which makes it possible to use CWP232291 in combination with other drugs, including those that exhibit such toxicity [116]. There is one phase 1 clinical trial of CWP232291 (NCT01398462).

PRI-724, an isomer of ICG-001, is a potent, specific inhibitor of the canonical Wnt signalling pathway in cancer stem cells with potential antineoplastic activity. PRI-724, like ICG-001, can inhibit the CBP/β-catenin interaction, thereby inhibiting the activity of the Wnt/β-catenin pathway in AML and CML [117]. There is one related phase 2 clinical trial (NCT01606579).

Overcoming drug resistance is important for cancer treatment. Cancer cells are less sensitive to traditional therapeutic drugs, resulting in difficulty in killing cancer cells as well as a significantly high recurrence rate. Pgp is a glycoprotein that is closely related to drug resistance in cancer and is a downstream target gene of Wnt signalling. In a study on ALL, Lawsone derivatives were found to target the Wnt/β-catenin signalling pathway in resistant leukaemia cells, increasing drug sensitivity in these resistant cells. The ability of Lawsone derivatives to bind to the CRD structural domains of FZD7 and FZD8 may stimulate internalization and degradation of Frizzled, blocking Wnt signalling transduction to the intracellular compartment and thus inhibiting Pgp expression [118].

Regarding therapies targeting the Wnt/β-catenin pathway in lymphoma and MM, there are currently no registered clinical trials. However, several new preclinical studies may reveal promising strategies for treatment.

WNT974, an inhibitor of the canonical Wnt pathway, is effective in the treatment of lymphoma because it posttranslationally induces defective endo-acylation of Wnt protein to block Wnt/β-catenin signalling, thus inhibiting cell proliferation, inducing apoptosis, and enhancing sensitivity to doxorubicin. These results suggest the potential of WNT974 as a therapeutic agent for lymphoma [119, 120].

Researchers have investigated the relationship between PRMT5 and Wnt/β-catenin signalling as well as AKT/GSK3β proliferative signalling in three different types of NHL cell lines, clinical samples, and mouse primary lymphoma cells. Chung et al. found that PRMT5 governs the expression of prosurvival genes by promoting Wnt/β-catenin and AKT/GSK3β proliferative signalling, and inhibition of PRMT5 induces lymphoma cell death; this strategy warrants further clinical evaluation [121].

In MM, the new Wnt canonical pathway inhibitor AV-65 has been shown to enhance ubiquitination and subsequent proteasomal degradation of β-catenin. The promoter activity of β-catenin/TCF target genes was also reduced. AV-65 successfully suppressed the progression of multiple myeloma in a mouse model [122].

The classical demethylating drug DAC combined with the protease inhibitor BZM has synergistic efficacy in the treatment of multiple myeloma via effects on proteins in the Wnt/β-catenin pathway. DAC reduced BZM-induced GSK3β (Ser9) phosphorylation and β-catenin accumulation in the nucleus, enhanced BZM-induced apoptosis, and promoted BZM-induced cell cycle arrest [123]. Further studies may optimize therapeutic regimens for MM.

BC2059 is a novel Wnt/beta-catenin pathway inhibitor. It can disrupt the binding of β-catenin to TBL1 and its related protein TBLR1, facilitating its destruction. BC2059 was confirmed to decrease β-catenin protein levels and the expression of downstream target genes. Furthermore, BC2059 was shown to synergize with low doses of the proteasomal inhibitor BZM in killing MM cells and was effective in a murine xenograft model of human MM, thus providing a rationale for further evaluation of the drug in the treatment of MM [124].

Panobinostat was approved by the FDA in 2015 for patients with relapsed MM. A recent study indicated that panobinostat and the β-catenin inhibitor Tegavivint had a favourable toxicity profile both in vitro and in vivo. Given the significant anti-MM effect of this novel combination, the strategy warrants further evaluation as a treatment for MM patients with relapsed and refractory MM [125].

## Conclusion

In conclusion, the role of the canonical Wnt signalling pathway in malignant haematologic diseases is unquestionable. There are various ways to modulate this pathway. Abnormal expression of Wnt ligands; loss of function due to mutations in factors that make up the destruction complex, such as APC and AXIN; and abnormalities in the activation and nuclear translocation of β-catenin can all contribute to cancer occurrence. Therapeutic approaches targeting Wnt/β-catenin are effective, and many treatment options have been mentioned in this article. However, these treatments have a variety of adverse effects. For example, CWP232291 treatment has been found to result in gastrointestinal symptoms (nausea, vomiting and diarrhoea), infusion-related reactions, and myalgia [126]. BC2059 also kills normal cells inevitably while killing MM cells. The combination of panobinostat and Tegavivint has been shown to have low toxicity, but even low toxicity can be harmful. It is still necessary to find new methods that are more efficacious and have fewer side effects.

## Data Availability

Not applicable.

## Abbreviations

APC: adenomatous polyposis coli
MASTL: microtubule-associated serine/threonine kinase-like
Linc00210: a long noncoding RNA
CTNNBIP1: recombinant human β-catenin-interacting protein 1
HCC: hepatocellular carcinoma
YAP: Yes-associated protein
TAZ: tafazzin
GRP78: heat shock protein
EMT: epithelial-mesenchymal transition
EBV: Epstein‒Barr virus
PLC: phospholipase C
PKC: protein kinase C
AXIN: a multidomain scaffold protein
GSK3β: glycogen synthase kinase 3β
CK1α: casein kinase 1α
PP2A: protein phosphatase 2A
FZD: Frizzled protein
CRD: cysteine-rich domain
PDZ: postsynaptic density-95/Discs large/Zonula occludens 1 domain
LRP5/6: low-density lipoprotein receptor-related protein 5/6
DVL: Dishevelled
TCF/LEF: T-cell factor/lymphoid enhancer-binding factor
HSC: haematopoietic stem cell
DKK: Dickkopf
CBP: CREB-binding protein
RUNX1: Runt-related transcription factor 1
LSCs: leukaemia stem cells
ALL: acute lymphoblastic leukaemia
P13K: phosphatidylinositol 3-kinase
AKT: protein kinase B
mTOR: mechanistic target of rapamycin
MYC: myelocytomatosis oncogene
AML: acute myeloid leukaemia
CML: chronic myelogenous leukaemia
FLT3: FMS-like tyrosine kinase 3
C-82: β-catenin inhibitor
DIXDC1: DIX domain-containing-1
CEBPA: CCAAT/enhancer binding protein-α
LncRNA: long noncoding RNA
FUT4: fucosyltransferase 4
FRAT2: frequently rearranged in advanced T-cell lymphomas 2
WIF-1: Wnt inhibitory factor 1
HL: Hodgkin’s lymphoma
NHL: non-Hodgkin’s lymphoma
DLBCL: diffuse large B-cell lymphoma
FIRRE: lncRNA functional intergenic repeat RNA elements
FOXM1: Forkhead box M1
TIMD4: T-cell immunoglobulin and mucin-containing structural domain 4
Foxp1: Forkhead box protein P1
FBN1: fibrillin-1
TIMP1: tissue inhibitor of metalloproteinase-1
GPNMB: glycoprotein nonmetastatic melanoma protein B
MM: multiple myeloma
SOX12: SRY-related high-mobility-group box 12
BCL9: B-cell CLL/lymphoma 9
ERK: extracellular signal-regulated kinase
MEK: mitogen-activated protein kinase kinase
PCDH10: protocadherin 10
ALDH: acetaldehyde dehydrogenase
MMSCs: marrow mesenchymal stem cells
BZM: bortezomib
Pgp: P-glycoprotein
PRMT5: protein arginine methyltransferase 5
DAC: decitabine
TBL1: transducin β-like protein 1
FDA: Food and Drug Administration
DHA: dihydroartemisinin

## References

[1] Rochford R, Cannon MJ, Moormann AM (2005). Endemic Burkitt’s lymphoma: a polymicrobial disease?. Nat Rev Microbiol.

[2] Gonzalez-Herrero I, Rodriguez-Hernandez G, Luengas-Martinez A, Isidro-Hernandez M, Jimenez R, Garcia-Cenador MB, et al. The Making of Leukemia. Int J Mol Sci. 2018;19(5). DOI:10.3390/ijms19051494. 10.3390/ijms19051494 PMC5983781 29772764

[3] Kumar SK, Rajkumar V, Kyle RA, van Duin M, Sonneveld P, Mateos MV (2017). Multiple myeloma. Nat Rev Dis Primers.

[4] Nusse R, Varmus HE (1992). Wnt genes. Cell.

[5] Vleminckx K, Wong E, Guger K, Rubinfeld B, Polakis P, Gumbiner BM (1997). Adenomatous polyposis coli tumor suppressor protein has signaling activity in Xenopus laevis embryos resulting in the induction of an ectopic dorsoanterior axis. J Cell Biol.

[6] Uppada SB, Gowrikumar S, Ahmad R, Kumar B, Szeglin B, Chen X (2018). MASTL induces Colon Cancer progression and Chemoresistance by promoting Wnt/beta-catenin signaling. Mol Cancer.

[7] Fu X, Zhu X, Qin F, Zhang Y, Lin J, Ding Y (2018). Linc00210 drives Wnt/beta-catenin signaling activation and liver tumor progression through CTNNBIP1-dependent manner. Mol Cancer.

[8] Kim W, Khan SK, Gvozdenovic-Jeremic J, Kim Y, Dahlman J, Kim H (2017). Hippo signaling interactions with Wnt/beta-catenin and Notch signaling repress liver tumorigenesis. J Clin Invest.

[9] Lager TW, Conner C, Keating CR, Warshaw JN, Panopoulos AD (2021). Cell surface GRP78 and Dermcidin cooperate to regulate breast cancer cell migration through Wnt signaling. Oncogene.

[10] He Y, Jiang X, Duan L, Xiong Q, Yuan Y, Liu P (2021). LncRNA PKMYT1AR promotes cancer stem cell maintenance in non-small cell lung cancer via activating Wnt signaling pathway. Mol Cancer.

[11] Peng Y, Xu Y, Zhang X, Deng S, Yuan Y, Luo X (2021). A novel protein AXIN1-295aa encoded by circAXIN1 activates the Wnt/beta-catenin signaling pathway to promote gastric cancer progression. Mol Cancer.

[12] Wei CY, Zhu MX, Yang YW, Zhang PF, Yang X, Peng R (2019). Downregulation of RNF128 activates Wnt/beta-catenin signaling to induce cellular EMT and stemness via CD44 and CTTN ubiquitination in melanoma. J Hematol Oncol.

[13] Katoh M. WNT and FGF gene clusters (review). Int J Oncol. 2002;21(6):1269-73. DOI. 12429977

[14] Gomez-Orte E, Saenz-Narciso B, Moreno S, Cabello J (2013). Multiple functions of the noncanonical Wnt pathway. Trends Genet.

[15] Niehrs C (2012). The complex world of WNT receptor signalling. Nat Rev Mol Cell Biol.

[16] Ozawa M, Baribault H, Kemler R (1989). The cytoplasmic domain of the cell adhesion molecule uvomorulin associates with three independent proteins structurally related in different species. EMBO J.

[17] Stamos JL, Weis WI (2013). The beta-catenin destruction complex. Cold Spring Harb Perspect Biol.

[18] Kimelman D, Xu W (2006). beta-catenin destruction complex: insights and questions from a structural perspective. Oncogene.

[19] Su Y, Fu C, Ishikawa S, Stella A, Kojima M, Shitoh K (2008). APC is essential for targeting phosphorylated beta-catenin to the SCFbeta-TrCP ubiquitin ligase. Mol Cell.

[20] Wu G, Xu G, Schulman BA, Jeffrey PD, Harper JW, Pavletich NP (2003). Structure of a beta-TrCP1-Skp1-beta-catenin complex: destruction motif binding and lysine specificity of the SCF(beta-TrCP1) ubiquitin ligase. Mol Cell.

[21] Tolwinski NS, Wehrli M, Rives A, Erdeniz N, DiNardo S, Wieschaus E (2003). Wg/Wnt signal can be transmitted through arrow/LRP5,6 and Axin independently of Zw3/Gsk3beta activity. Dev Cell.

[22] Cong F, Schweizer L, Varmus H (2004). Wnt signals across the plasma membrane to activate the beta-catenin pathway by forming oligomers containing its receptors, Frizzled and LRP. Development.

[23] MacDonald BT, He X. Frizzled and LRP5/6 receptors for Wnt/beta-catenin signaling. Cold Spring Harb Perspect Biol. 2012;4(12). DOI:10.1101/cshperspect.a007880. 10.1101/cshperspect.a007880 PMC3504444 23209147

[24] Kishida S, Yamamoto H, Hino S, Ikeda S, Kishida M, Kikuchi A (1999). DIX domains of Dvl and axin are necessary for protein interactions and their ability to regulate beta-catenin stability. Mol Cell Biol.

[25] MacDonald BT, Yokota C, Tamai K, Zeng X, He X (2008). Wnt signal amplification via activity, cooperativity, and regulation of multiple intracellular PPPSP motifs in the Wnt co-receptor LRP6. J Biol Chem.

[26] Bilic J, Huang YL, Davidson G, Zimmermann T, Cruciat CM, Bienz M (2007). Wnt induces LRP6 signalosomes and promotes dishevelled-dependent LRP6 phosphorylation. Science.

[27] Davidson G, Wu W, Shen J, Bilic J, Fenger U, Stannek P (2005). Casein kinase 1 gamma couples Wnt receptor activation to cytoplasmic signal transduction. Nature.

[28] Mao J, Wang J, Liu B, Pan W, Farr GH, Flynn C (2001). Low-density lipoprotein receptor-related protein-5 binds to Axin and regulates the canonical Wnt signaling pathway. Mol Cell.

[29] MacDonald BT, Tamai K, He X (2009). Wnt/beta-catenin signaling: components, mechanisms, and diseases. Dev Cell.

[30] Chae WJ, Bothwell ALM (2018). Canonical and Non-Canonical Wnt Signaling in Immune Cells. Trends Immunol.

[31] Zhan T, Rindtorff N, Boutros M (2017). Wnt signaling in cancer. Oncogene.

[32] Eaves CJ (2015). Hematopoietic stem cells: concepts, definitions, and the new reality. Blood.

[33] Kwarteng EO, Hetu-Arbour R, Heinonen KM (2018). Frontline Science: Wnt/beta-catenin pathway promotes early engraftment of fetal hematopoietic stem/progenitor cells. J Leukoc Biol.

[34] Nemeth MJ, Mak KK, Yang Y, Bodine DM (2009). beta-Catenin expression in the bone marrow microenvironment is required for long-term maintenance of primitive hematopoietic cells. Stem Cells.

[35] Glinka A, Wu W, Delius H, Monaghan AP, Blumenstock C, Niehrs C (1998). Dickkopf-1 is a member of a new family of secreted proteins and functions in head induction. Nature.

[36] Staal FJ, Luis TC, Tiemessen MM (2008). WNT signalling in the immune system: WNT is spreading its wings. Nat Rev Immunol.

[37] Fleming HE, Janzen V, Lo Celso C, Guo J, Leahy KM, Kronenberg HM (2008). Wnt signaling in the niche enforces hematopoietic stem cell quiescence and is necessary to preserve self-renewal in vivo. Cell Stem Cell.

[38] Luis TC, Weerkamp F, Naber BA, Baert MR, de Haas EF, Nikolic T (2009). Wnt3a deficiency irreversibly impairs hematopoietic stem cell self-renewal and leads to defects in progenitor cell differentiation. Blood.

[39] Luis TC, Naber BA, Fibbe WE, van Dongen JJ, Staal FJ (2010). Wnt3a nonredundantly controls hematopoietic stem cell function and its deficiency results in complete absence of canonical Wnt signaling. Blood.

[40] Luis TC, Naber BA, Roozen PP, Brugman MH, de Haas EF, Ghazvini M (2011). Canonical wnt signaling regulates hematopoiesis in a dosage-dependent fashion. Cell Stem Cell.

[41] Wang Y, Cui H, Tao S, Zeng T, Wu J, Tao Z (2020). High Canonical Wnt/beta-Catenin Activity Sensitizes Murine Hematopoietic Stem and Progenitor Cells to DNA Damage. Stem Cell Rev Rep.

[42] Iyer NG, Ozdag H, Caldas C (2004). p300/CBP and cancer. Oncogene.

[43] Ma H, Nguyen C, Lee KS, Kahn M (2005). Differential roles for the coactivators CBP and p300 on TCF/beta-catenin-mediated survivin gene expression. Oncogene.

[44] Kawasaki H, Eckner R, Yao TP, Taira K, Chiu R, Livingston DM (1998). Distinct roles of the co-activators p300 and CBP in retinoic-acid-induced F9-cell differentiation. Nature.

[45] Fernandez JG, Rodriguez DA, Valenzuela M, Calderon C, Urzua U, Munroe D (2014). Survivin expression promotes VEGF-induced tumor angiogenesis via PI3K/Akt enhanced beta-catenin/Tcf-Lef dependent transcription. Mol Cancer.

[46] Wu C, Gupta N, Huang YH, Zhang HF, Alshareef A, Chow A (2018). Oxidative stress enhances tumorigenicity and stem-like features via the activation of the Wnt/beta-catenin/MYC/Sox2 axis in ALK-positive anaplastic large-cell lymphoma. BMC Cancer.

[47] Yin S, Li J, Hu C, Chen X, Yao M, Yan M (2007). CD133 positive hepatocellular carcinoma cells possess high capacity for tumorigenicity. Int J Cancer.

[48] Huang EH, Hynes MJ, Zhang T, Ginestier C, Dontu G, Appelman H (2009). Aldehyde dehydrogenase 1 is a marker for normal and malignant human colonic stem cells (SC) and tracks SC overpopulation during colon tumorigenesis. Cancer Res.

[49] Ginestier C, Hur MH, Charafe-Jauffret E, Monville F, Dutcher J, Brown M (2007). ALDH1 is a marker of normal and malignant human mammary stem cells and a predictor of poor clinical outcome. Cell Stem Cell.

[50] Landen CN, Goodman B, Katre AA, Steg AD, Nick AM, Stone RL (2010). Targeting aldehyde dehydrogenase cancer stem cells in ovarian cancer. Mol Cancer Ther.

[51] Su Y, Qiu Q, Zhang X, Jiang Z, Leng Q, Liu Z (2010). Aldehyde dehydrogenase 1 A1-positive cell population is enriched in tumor-initiating cells and associated with progression of bladder cancer. Cancer Epidemiol Biomarkers Prev.

[52] van den Hoogen C, van der Horst G, Cheung H, Buijs JT, Lippitt JM, Guzman-Ramirez N (2010). High aldehyde dehydrogenase activity identifies tumor-initiating and metastasis-initiating cells in human prostate cancer. Cancer Res.

[53] Lapidot T, Sirard C, Vormoor J, Murdoch B, Hoang T, Caceres-Cortes J (1994). A cell initiating human acute myeloid leukaemia after transplantation into SCID mice. Nature.

[54] Cancer: Principles & Practice of Oncology (9th ed.) (2011). - Medical Journal of Peking Union Medical College Hospital. 2012;- 3(- 3):- 357. DOI: -.

[55] Wang Y, Krivtsov AV, Sinha AU, North TE, Goessling W, Feng Z (2010). The Wnt/beta-catenin pathway is required for the development of leukemia stem cells in AML. Science.

[56] Medina MA, Ugarte GD, Vargas MF, Avila ME, Necunir D, Elorza AA (2016). Alternative RUNX1 Promoter Regulation by Wnt/beta-Catenin Signaling in Leukemia Cells and Human Hematopoietic Progenitors. J Cell Physiol.

[57] Yu H, Gao R, Chen S, Liu X, Wang Q, Cai W (2021). Bmi1 Regulates Wnt Signaling in Hematopoietic Stem and Progenitor Cells. Stem Cell Reviews and Reports.

[58] Perry JM, Tao F, Roy A, Lin T, He XC, Chen S (2020). Overcoming Wnt-beta-catenin dependent anticancer therapy resistance in leukaemia stem cells. Nat Cell Biol.

[59] Jin F, Wu Z, Hu X, Zhang J, Gao Z, Han X (2019). The PI3K/Akt/GSK-3beta/ROS/eIF2B pathway promotes breast cancer growth and metastasis via suppression of NK cell cytotoxicity and tumor cell susceptibility. Cancer Biol Med.

[60] Maynard MA, Ohh M (2007). The role of hypoxia-inducible factors in cancer. Cell Mol Life Sci.

[61] Kaidi A, Williams AC, Paraskeva C (2007). Interaction between β-catenin and HIF-1 promotes cellular adaptation to hypoxia. Nat Cell Biol.

[62] Giambra V, Jenkins CE, Lam SH, Hoofd C, Belmonte M, Wang X (2015). Leukemia stem cells in T-ALL require active Hif1α and Wnt signaling. Blood.

[63] Alvarez-Tejado M, Naranjo-Suarez S, Jimenez C, Carrera AC, Landazuri MO, del Peso L (2001). Hypoxia induces the activation of the phosphatidylinositol 3-kinase/Akt cell survival pathway in PC12 cells: protective role in apoptosis. J Biol Chem.

[64] Evangelisti C, Chiarini F, Cappellini A, Paganelli F, Fini M, Santi S (2020). Targeting Wnt/beta-catenin and PI3K/Akt/mTOR pathways in T-cell acute lymphoblastic leukemia. J Cell Physiol.

[65] Dang CV (2012). MYC on the path to cancer. Cell.

[66] Hara K, Kageji T, Mizobuchi Y, Kitazato KT, Okazaki T, Fujihara T (2015). Blocking of the interaction between Wnt proteins and their co-receptors contributes to the anti-tumor effects of adenovirus-mediated DKK3 in glioblastoma. Cancer Lett.

[67] Giralt I, Gallo-Oller G, Navarro N, Zarzosa P, Pons G, Magdaleno A, et al. Dickkopf Proteins and Their Role in Cancer: A Family of Wnt Antagonists with a Dual Role. Pharmaceuticals. 2021;14(8). DOI:10.3390/ph14080810. 10.3390/ph14080810 PMC8400703 34451907

[68] Kong D, Zhao L, Sun L, Fan S, Li H, Zhao Y (2018). MYCN is a novel oncogenic target in adult B-ALL that activates the Wnt/beta-catenin pathway by suppressing DKK3. J Cell Mol Med.

[69] Papaemmanuil E, Gerstung M, Bullinger L, Gaidzik VI, Paschka P, Roberts ND (2016). Genomic Classification and Prognosis in Acute Myeloid Leukemia. N Engl J Med.

[70] Jiang X, Mak PY, Mu H, Tao W, Mak DH, Kornblau S (2018). Disruption of Wnt/beta-Catenin Exerts Antileukemia Activity and Synergizes with FLT3 Inhibition in FLT3-Mutant Acute Myeloid Leukemia. Clin Cancer Res.

[71] Almars A, Chondrou PS, Onyido EK, Almozyan S, Seedhouse C, Babaei-Jadidi R, et al. Increased FLYWCH1 Expression is Negatively Correlated with Wnt/beta-catenin Target Gene Expression in Acute Myeloid Leukemia Cells. Int J Mol Sci. 2019;20(11). DOI:10.3390/ijms20112739. 10.3390/ijms20112739 PMC6600431 31167387

[72] Shiomi K, Uchida H, Keino-Masu K, Masu M (2003). Ccd1, a novel protein with a DIX domain, is a positive regulator in the Wnt signaling during zebrafish neural patterning. Curr Biol.

[73] Xin H, Li C, Wang M (2018). DIXDC1 promotes the growth of acute myeloid leukemia cells by upregulating the Wnt/beta-catenin signaling pathway. Biomed Pharmacother.

[74] Sun C, Luan S, Zhang G, Wang N, Shao H, Luan C. CEBPA-mediated upregulation of the lncRNA PLIN2 promotes the development of chronic myelogenous leukemia via the GSK3 and Wnt/beta-catenin signaling pathways. Am J Cancer Res. 2017;7(5):1054-67. DOI. PMC5446474 28560057

[75] Szwed M, Kania KD, Jozwiak Z (2015). Toxicity of doxorubicin-transferrin conjugate is connected to the modulation of Wnt/beta-catenin pathway in human leukemia cells. Leuk Res.

[76] Ruan X, Jiang W, Cheng P, Huang L, Li X, He Y (2018). Volatile anesthetics sevoflurane targets leukemia stem/progenitor cells via Wnt/beta-catenin inhibition. Biomed Pharmacother.

[77] Li Y, Hu J, Song H, Wu T (2018). Antibiotic anisomycin selectively targets leukemia cell lines and patient samples through suppressing Wnt/beta-catenin signaling. Biochem Biophys Res Commun.

[78] Chiarini F, Paganelli F, Martelli AM, Evangelisti C. The Role Played by Wnt/beta-Catenin Signaling Pathway in Acute Lymphoblastic Leukemia. Int J Mol Sci. 2020;21(3). DOI:10.3390/ijms21031098. 10.3390/ijms21031098 PMC7037748 32046053

[79] Li J, Hsu HC, Mountz JD, Allen JG (2018). Unmasking Fucosylation: from Cell Adhesion to Immune System Regulation and Diseases. Cell Chem Biol.

[80] Zhang Y, Chen HX, Zhou SY, Wang SX, Zheng K, Xu DD (2015). Sp1 and c-Myc modulate drug resistance of leukemia stem cells by regulating survivin expression through the ERK-MSK MAPK signaling pathway. Mol Cancer.

[81] Chen JJ, Lei P, Zhou M (2020). hsa_circ_0121582 inhibits leukemia growth by dampening Wnt/beta-catenin signaling. Clin Transl Oncol.

[82] van Amerongen R, van der Gulden H, Bleeker F, Jonkers J, Berns A (2004). Characterization and functional analysis of the murine Frat2 gene. J Biol Chem.

[83] Li N, Ma Y, Wang W, Yin CC, Wu W, Sun R (2020). LOC101928834, a novel lncRNA in Wnt/beta-catenin signaling pathway, promotes cell proliferation and predicts poor clinical outcome in myelodysplastic syndromes. Clin Sci (Lond).

[84] Li GJ, Ding H, Miao D (2019). Long-noncoding RNA HOTAIR inhibits immunologic rejection of mouse leukemia cells through activating the Wnt/beta-catenin signaling pathway in a mouse model of leukemia. J Cell Physiol.

[85] Ge XS, Ma HJ, Zheng XH, Ruan HL, Liao XY, Xue WQ (2013). HOTAIR, a prognostic factor in esophageal squamous cell carcinoma, inhibits WIF-1 expression and activates Wnt pathway. Cancer Sci.

[86] Zhao CC, Jiao Y, Zhang YY, Ning J, Zhang YR, Xu J (2019). Lnc SMAD5-AS1 as ceRNA inhibit proliferation of diffuse large B cell lymphoma via Wnt/beta-catenin pathway by sponging miR-135b-5p to elevate expression of APC. Cell Death Dis.

[87] Magalhaes L, Quintana LG, Lopes DCF, Vidal AF, Pereira AL, D’Araujo Pinto LC (2018). APC gene is modulated by hsa-miR-135b-5p in both diffuse and intestinal gastric cancer subtypes. BMC Cancer.

[88] Hu Y, Zhao Y, Shi C, Ren P, Wei B, Guo Y (2019). A circular RNA from APC inhibits the proliferation of diffuse large B-cell lymphoma by inactivating Wnt/beta-catenin signaling via interacting with TET1 and miR-888. Aging.

[89] Vockerodt M, Yap LF, Shannon-Lowe C, Curley H, Wei W, Vrzalikova K (2015). The Epstein-Barr virus and the pathogenesis of lymphoma. J Pathol.

[90] Zhao CX, Yan ZX, Wen JJ, Fu D, Xu PP, Wang L (2021). CircEAF2 counteracts Epstein-Barr virus-positive diffuse large B-cell lymphoma progression via miR-BART19-3p/APC/beta-catenin axis. Mol Cancer.

[91] Shi X, Cui Z, Liu X, Wu S, Wu Y, Fang F (2019). LncRNA FIRRE is activated by MYC and promotes the development of diffuse large B-cell lymphoma via Wnt/beta-catenin signaling pathway. Biochem Biophys Res Commun.

[92] Meng H, Zhao B, Wang Y (2020). FOXM1-induced upregulation of lncRNA OR3A4 promotes the progression of diffuse large B-cell lymphoma via Wnt/beta-catenin signaling pathway. Exp Mol Pathol.

[93] Li Y, Zhang PY, Yang ZW, Ma F, Li FX (2020). TIMD4 exhibits regulatory capability on the proliferation and apoptosis of diffuse large B-cell lymphoma cells via the Wnt/beta-catenin pathway. J Gene Med.

[94] Walker MP, Stopford CM, Cederlund M, Fang F, Jahn C, Rabinowitz AD (2015). FOXP1 potentiates Wnt/beta-catenin signaling in diffuse large B cell lymphoma. Sci Signal.

[95] Wang H, Liu Z, Zhang G. FBN1 promotes DLBCL cell migration by activating the Wnt/beta-catenin signaling pathway and regulating TIMP1. Am J Transl Res. 2020;12(11):7340-53. DOI. PMC7724331 33312371

[96] Weterman MA, Ajubi N, van Dinter IM, Degen WG, van Muijen GN, Ruitter DJ (1995). nmb, a novel gene, is expressed in low-metastatic human melanoma cell lines and xenografts. Int J Cancer.

[97] Wang Z, Ran X, Qian S, Hou H, Dong M, Wu S (2021). GPNMB promotes the progression of diffuse large B cell lymphoma via YAP1-mediated activation of the Wnt/beta-catenin signaling pathway. Arch Biochem Biophys.

[98] Zhou H, Tang H, Li N, Chen H, Chen X, Gu L (2020). MicroRNA-361-3p Inhibit the Progression of Lymphoma by the Wnt/beta-Catenin Signaling Pathway. Cancer Manag Res.

[99] Palumbo A, Anderson K (2011). Multiple myeloma. N Engl J Med.

[100] Zhang W, Yu F, Weng J, Zheng Y, Lin J, Qi T (2021). SOX12 Promotes Stem Cell-Like Phenotypes and Osteosarcoma Tumor Growth by Upregulating JAGGED1. Stem Cells Int.

[101] Du F, Feng W, Chen S, Wu S, Cao T, Yuan T (2019). Sex determining region Y-box 12 (SOX12) promotes gastric cancer metastasis by upregulating MMP7 and IGF1. Cancer Lett.

[102] Gao Y, Li L, Hou L, Niu B, Ru X, Zhang D (2020). SOX12 promotes the growth of multiple myeloma cells by enhancing Wnt/beta-catenin signaling. Exp Cell Res.

[103] Guo B, Xiao C, Liu Y, Zhang N, Bai H, Yang T (2021). miR-744-5p Inhibits Multiple Myeloma Proliferation, Epithelial Mesenchymal Transformation and Glycolysis by Targeting SOX12/Wnt/beta-Catenin Signaling. Onco Targets Ther.

[104] Yang Y, Li Y, Wang Y, Wu J, Yang G, Yang T (2012). Versican gene: regulation by the beta-catenin signaling pathway plays a significant role in dermal papilla cell aggregative growth. J Dermatol Sci.

[105] Chen H, Zhao Y, Zhang J, Xie Y, Jin M (2021). Promoting effects of MiR-135b on human multiple myeloma cells via regulation of the Wnt/beta-catenin/Versican signaling pathway. Cytokine.

[106] Zhao JJ, Lin J, Zhu D, Wang X, Brooks D, Chen M (2014). miR-30-5p functions as a tumor suppressor and novel therapeutic tool by targeting the oncogenic Wnt/beta-catenin/BCL9 pathway. Cancer Res.

[107] Zhou F, Wang D, Zhou N, Chen H, Shi H, Peng R (2021). Circular RNA Protein Tyrosine Kinase 2 Promotes Cell Proliferation, Migration and Suppresses Apoptosis via Activating MicroRNA-638 Mediated MEK/ERK, WNT/beta-Catenin Signaling Pathways in Multiple Myeloma. Front Oncol.

[108] Wu L, Zhou Z, Han S, Chen J, Liu Z, Zhang X (2020). PLAGL2 promotes epithelial-mesenchymal transition and mediates colorectal cancer metastasis via beta-catenin-dependent regulation of ZEB1. Br J Cancer.

[109] Liu Q, Ran R, Song M, Li X, Wu Z, Dai G (2021). LncRNA HCP5 acts as a miR-128-3p sponge to promote the progression of multiple myeloma through activating Wnt/beta-catenin/cyclin D1 signaling via PLAGL2. Cell Biol Toxicol.

[110] Liu X, Peng J, Zhou Y, Xie B, Wang J (2019). Silencing RRM2 inhibits multiple myeloma by targeting the Wnt/betacatenin signaling pathway. Mol Med Rep.

[111] Xu Y, Yang Z, Yuan H, Li Z, Li Y, Liu Q (2015). PCDH10 inhibits cell proliferation of multiple myeloma via the negative regulation of the Wnt/beta-catenin/BCL-9 signaling pathway. Oncol Rep.

[112] Wang H, Gong Y, Liang L, Xiao L, Yi H, Ye M (2020). Lycorine targets multiple myeloma stem cell-like cells by inhibition of Wnt/beta-catenin pathway. Br J Haematol.

[113] Wu X, Liu Y, Zhang E, Chen J, Huang X, Yan H (2020). Dihydroartemisinin Modulates Apoptosis and Autophagy in Multiple Myeloma through the P38/MAPK and Wnt/beta-Catenin Signaling Pathways. Oxid Med Cell Longev.

[114] Geng W, Guo X, Zhang L, Ma Y, Wang L, Liu Z (2018). Resveratrol inhibits proliferation, migration and invasion of multiple myeloma cells via NEAT1-mediated Wnt/beta-catenin signaling pathway. Biomed Pharmacother.

[115] Choi PJ, O Y, Her JH, Yun E, Song GY, Oh S (2017). Anti-proliferative activity of CGK012 against multiple myeloma cells via Wnt/beta-catenin signaling attenuation. Leuk Res.

[116] Cortes JE, Kim M, Lee K, Choi J, Lee H, Becker PS (2020). Phase 1 study of CWP232291 in patients with relapsed or refractory acute myeloid leukemia and myelodysplastic syndrome. Blood Adv.

[117] Nishikawa K, Osawa Y, Kimura K. Wnt/β-Catenin Signaling as a Potential Target for the Treatment of Liver Cirrhosis Using Antifibrotic Drugs. Int J Mol Sci. 2018;19(10). DOI:10.3390/ijms19103103. 10.3390/ijms19103103 PMC6213128 30308992

[118] Hamdoun S, Fleischer E, Klinger A, Efferth T (2017). Lawsone derivatives target the Wnt/beta-catenin signaling pathway in multidrug-resistant acute lymphoblastic leukemia cells. Biochem Pharmacol.

[119] Chen S, Yuan X, Xu H, Yi M, Liu S, Wen F (2020). WNT974 Inhibits Proliferation, Induces Apoptosis, and Enhances Chemosensitivity to Doxorubicin in Lymphoma Cells by Inhibiting Wnt/beta-Catenin Signaling. Med Sci Monit.

[120] Takada R, Satomi Y, Kurata T, Ueno N, Norioka S, Kondoh H (2006). Monounsaturated fatty acid modification of Wnt protein: its role in Wnt secretion. Dev Cell.

[121] Chung J, Karkhanis V, Baiocchi RA, Sif S (2019). Protein arginine methyltransferase 5 (PRMT5) promotes survival of lymphoma cells via activation of WNT/β-catenin and AKT/GSK3β proliferative signaling. J Biol Chem.

[122] Yao H, Ashihara E, Strovel JW, Nakagawa Y, Kuroda J, Nagao R (2011). AV-65, a novel Wnt/beta-catenin signal inhibitor, successfully suppresses progression of multiple myeloma in a mouse model. Blood Cancer J.

[123] Jin Y, Xu L, Wu X, Feng J, Shu M, Gu H (2019). Synergistic Efficacy of the Demethylation Agent Decitabine in Combination With the Protease Inhibitor Bortezomib for Treating Multiple Myeloma Through the Wnt/beta-Catenin Pathway. Oncol Res.

[124] Savvidou I, Khong T, Cuddihy A, McLean C, Horrigan S, Spencer A. beta-Catenin Inhibitor BC2059 Is Efficacious as Monotherapy or in Combination with Proteasome Inhibitor Bortezomib in Multiple Myeloma. Mol Cancer Ther. 2017;16(9):1765-78. DOI: 10.1158/1535-7163.MCT-16-0624. 10.1158/1535-7163.MCT-16-0624 28500235

[125] Savvidou I, Khong T, Whish S, Carmichael I, Sepehrizadeh T, Mithraprabhu S, et al. Combination of Histone Deacetylase Inhibitor Panobinostat (LBH589) with beta-Catenin Inhibitor Tegavivint (BC2059) Exerts Significant Anti-Myeloma Activity Both In Vitro and In Vivo. Cancers (Basel). 2022;14(3). DOI: 10.3390/cancers14030840. 10.3390/cancers14030840 PMC8834319 35159107

[126] Lee JH, Faderl S, Pagel JM, Jung CW, Yoon SS, Pardanani AD (2020). Phase 1 study of CWP232291 in patients with relapsed or refractory acute myeloid leukemia and myelodysplastic syndrome. Blood Adv.

